# Surface Finishing of FDM-Fabricated Amorphous Polyetheretherketone and Its Carbon-Fiber-Reinforced Composite by Dry Milling

**DOI:** 10.3390/polym13132175

**Published:** 2021-06-30

**Authors:** Cheng Guo, Xiaohua Liu, Guang Liu

**Affiliations:** 1 College of Mechatronics and Control Engineering, Shenzhen University, Shenzhen 518060, China; cheng.guo@szu.edu.cn (C.G.); liuxh89@126.com (X.L.); 2 College of Physics and Optoelectronic Engineering, Shenzhen University, Shenzhen 518060, China; 3 School of System Design and Intelligent Manufacturing, Southern University of Science and Technology, Shenzhen 518055, China

**Keywords:** PEEK, CF/PEEK, FDM, dry milling, hybrid additive-subtractive manufacturing

## Abstract

In recent years, many investigations have been devoted to fused deposition modeling (FDM) of high-performance polymer-polyetheretherketone (PEEK) and carbon-fiber-reinforced PEEK (CF/PEEK) for biomedical and aerospace applications. However, the staircase effect naturally brought about by FDM restricts further applications of 3D-printed PEEK and its composites in high-temperature molds, medical implants, and precision components, which require better or customized surface qualities. Hence, this work aimed to reduce the staircase effect and improve the surface quality of 3D-printed PEEK and CF/PEEK parts by dry milling of the fluctuant exterior surface. The co-dependency between 3D printing parameters (raster angle and layer thickness) and milling parameters (depth of cut, spindle speed, and feed rate per tooth) were investigated through experiments. The difference in removal mechanisms for PEEK and CF/PEEK was revealed. It was confirmed that the smearing effect enhanced the surface quality based on the morphology analysis and the simulation model. Both the raster angle of +45°/−45° and the small layer thickness could improve the surface quality of these 3D-printed polymers after dry milling. A large depth of cut and a large feed rate per tooth were likely to deteriorate the finished polymer surface. The spindle speed could influence the morphologies without significant changes in roughness values. Finally, a demonstration was performed to verify that dry milling of 3D-printed amorphous PEEK and CF/PEEK parts could lead to a high surface quality for critical requirements.

## 1. Introduction

Because of the combination of excellent chemical resistance, thermal stability, and mechanical properties [1,2,3], polyetheretherketone (PEEK), a kind of semi-crystalline polymer, and its composites have attracted a great deal of interest from both scientists and engineers [4]. These kinds of polymers possess great potential to replace conventional metal materials because of their high strength-to-weight ratios, especially for the aviation and space industry [5]. In addition, because of their good biochemical properties and radio transparency [6,7], PEEK and its composites have been increasingly employed in medical implants, such as dental screws [8] and bone replacement [9].

Because of the high cost and versatile usage scenarios of PEEK parts, investigations on manufacturing methods for PEEK components are still in the ascendant. L. Romoliet et al. [10] conducted an investigation on laser drilling of PEEK reinforced with carbon fibers and achieved holes with diameters of 2 mm and 0.1 mm. The laser-based technique is still subject to the heat-affected zone (HAZ). In the domain of cutting technologies, I. Hanafi et al. [11] reported that the cutting speed and the depth of cut (DOC) were the most influential parameters when dry machining carbon-fiber-reinforced PEEK (PEEK-CF30) with TiN coated tools. J. Paulo Davim et al. built up a cutting model of PEEK composites [12] and examined the performance of CVD diamond-coated tools [13] and PCD tools [14] in machining PEEK composites. They verified that it was possible to obtain IT 6 precision for components made of PEEK and its composites with appropriate cutting parameters [15]. D. Kim et al. [16] evaluated the influence of the consolidation process on the machinability of PEEK thermoplastic composites. They found that the induction-processed composite material produced equivalent or better holes than the autoclave-processed composites. J. Xu et al. [17] compared the drilling performance of carbon/PEEK and carbon/polyimide (PI) composites. They confirmed that when drilling the carbon/PEEK composites, higher drilling forces and higher cutting temperature occurred compared with carbon/polyimide composites, indicating poor machinability. M. Khoran [18] utilized cryogenic cooling to improve the surface quality and to reduce the tool loading when grinding PEEK. In addition to the turning and drilling of PEEK and related materials, many works focused on the milling of PEEK, because the milling operation can achieve complex 3D shapes. R. Izamshah et al. [19] studied the surface roughness of unfilled PEEK engineering plastics after milling. It was found that the feed rate (FR) was the main factor that influenced the surface roughness followed by the spindle speed (SS) and the DOC. Some form of polymeric softening occurred when the cutting speed exceeded a critical cutting speed. H. Cao et al. [20] optimized the process of high-speed dry milling unidirectional (UD) CF/PEEK based on a genetic algorithm BP neural network. They found that the cutting speed and the fiber orientation were significant factors affecting the machined surface microstructural characteristics. Compared with metals, polymers are relatively soft, inducing manufacturing problems related to machining and deburring.

Compared with the above-mentioned subtractive manufacturing methods for PEEK and its composites, three-dimensional (3D) printing techniques, such as fused deposition modeling (FDM), gets around many restraints and is very suitable for the fabrication of small-batch customized PEEK components because of the excellent heat resistance of PEEK and its composites [21,22,23]. M. Yan et al. [21] examined the rheological behavior during selective laser sintering (SLS) and the sintering kinetics of CF/PEEK composites, paving the way for the structure optimization and light-weight design with complex geometries. More investigations were based on fused filaments. For example, Krzysztof Rodzeń et al. [24] attempted to directly 3D print functional PEEK/hydroxyapatite composites with fused filaments. C. Yang et al. [25] proposed a temperature-controlled 3D printing method for PEEK, which can realize different degrees of crystallinity and mechanical properties for customized PEEK parts. M. Luo et al. [26] paid attention to a laser-assisted material extrusion process for PEEK, which effectively improved both the interlayer shear strength and the crystallinity simultaneously. P. Wang et al. [27] presented a constitutive equation considering the dynamic viscosity for PEEK during FDM using finite element analysis (FEA). A.E. Magri et al. [28] confirmed that the nozzle temperature was the most influential parameter on tensile properties and the crystallinity degree of printed PEEK. They also verified that annealing of the PEEK parts after 3D printing can further improve tensile properties by enhancing the crystalline structure of the PEEK chains.

However, some issues still restrict utilization of 3D-printed PEEK components. For example, the surface roughness is vital for medical implants made of PEEK, because cells or tissues directly interact with the PEEK surface on a very small scale. In addition, direct printing circuits or electronics on 3D-printed parts, i.e., hybrid 3D printing, has been extensively investigated in recent years [29,30]. A good surface finishing of 3D-printed parts is crucial to multifunctional structures with printed electronics. Currently, one major challenge of hybrid 3D printing is the poor surface morphology of 3D-printed parts, resulting in poor printability of the conductive ink [31]. Hence, considering the large surface roughness induced by 3D printing, additional smoothing operations are required [32,33]. In recent years, the hybrid additive–subtractive manufacturing process has been put forward [34,35] to efficiently and effectively remove the 3D-printing-induced defects and improve the surface quality. For instance, L. Li et al. [36] proposed a novel six-axis hybrid manufacturing process to remove the staircase error, which was based on a six-degrees-of-freedom robot arm equipped with multiple changeable heads (printing head and milling head). This scheme improved the surface quality and reduced material waste for the production of high-performance polymer parts. Compared to media blasting and liquid chemical treatment, dry milling could avoid the introduction of additional contaminants. Simultaneously, dry milling is capable to increase the precision of the 3D-printed parts with simple operations.

In this work, in order to meet the critical requirements of 3D-printed molds, special mating surfaces, and biomedical implants, an investigation on dry milling of the exterior stepped surfaces of the amorphous PEEK and CF/PEEK parts fabricated by FDM was performed. After this operation, heat treatment can be conducted to increase crystallinity. To be specific, the printing parameters: raster angles (RA) and layer thickness (LT), which are always selected to derive customized anisotropic mechanical performance, were evaluated in terms of roughness after dry milling. The milling parameters: spindle speed (SS), depth of cut (DOC), and feed rate per tooth (FRT) were also examined to assess their effects on the surface quality. The smearing effect and cavity formation were analyzed by experiments and a simulation model. Finally, a demonstration of dry milling 3D-printed PEEK and CF/PEEK parts with optimized parameters has been illustrated to verify the usability of the post-processing method for 3D-printed amorphous polymers.

## 2. Materials and Methods

### 2.1. Materials and Material Handling Equipment

**Figure 1:**
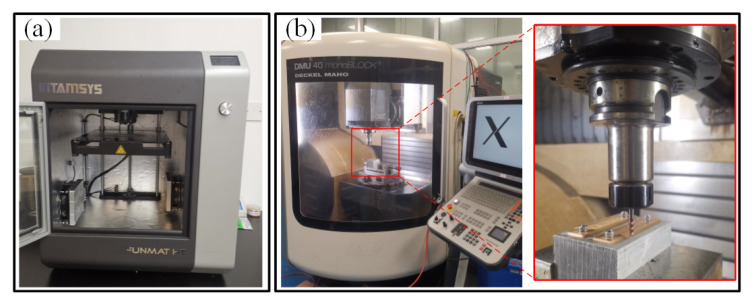
Material handling equipment: (**a**) FUNMAT HT 3D printer; (**b**) DMU40 machining center.

A FUNMAT HT desktop industrial 3D printer (INTAMSYS, Shanghai, China) was utilized to produce the 3D-printed PEEK and CF/PEEK parts, as shown in Figure 1a. Its advanced thermal design was very suitable for PEEK and other high-performance functional materials. Filaments of PEEK and CF/PEEK with a diameter of 1.75 mm were also provided by the same company. The diameter of CF was 5~10μm, and the content of CF was 10 wt.%. The mechanical properties of these materials are listed in Table 1. Before printing, filaments were dried for 5 h under 150 °C. A five-axis machining center (Figure 1b), DMU40 (DMG MORI, Hamburg, Germany), was adopted for the milling operation to investigate the machining surface roughness with respect to the changes in the DOC, the SS, the FRT, etc. The maximum SS could be up to 20,000 r/min. The machine precision was +/−2μm. The embedded numerical control system was HEIDENHAIN ITNC 530 (HEIDENHAIN, Berlin, Germany). Registration of the polymer sample to be milled was achieved by a built-in contact probe. The cutting tool was an integral end mill made of cemented carbide, with 4 TiSiN coated edges (Luhao, Jinhua, China), as shown in the enlarged view of Figure 1b. The particle size of the tungsten carbide was ca. 0.6 μm and the diameter of the end mill was 4 mm.

### 2.2. Preparation of the 3D-Printed Samples

The geometric models for 3D printing were designed in UG NX software, and the exported files (stl) were fed into a built-in INTAM-SUITE software, in charge of model slicing and parameter setting. The printing parameters and conditions are listed in Table 2. The nozzle temperature was set to 400 °C, a recommended temperature from the equipment company. The PEEK and CF/PEEK samples for the milling experiments were designed into blocks with the dimensions of 80 mm × 20 mm × 2 mm, which can be mounted on the fixture as illustrated in Figure 1b. The 3D-printed samples were fabricated without further thermal treatment. Several typical values of the LT and the RA were selected for preparation of the 3D-printed samples.

### 2.3. Test Equipment

The scanning electron microscope (SEM), Quanta FEG 450 (FEI, Hillsboro, OR, USA), was used for observing microstructures of the finished surface. The laser scanning confocal microscope (LSCM), VK-X2000 (KEYENCE, Osaka, Japan), was utilized to measure the surface roughness and burr formation. The dimensions of all the 3D colorful morphology micrographs in the following part captured by this LSCM were 1062 μm × 1417 μm. X-ray diffraction (XRD) (MiniFlex600, Rigaku, Tokyo, Japan) was used for characterization of the crystallinity of the samples. The diffraction angle (2θ) range was set to 2~50°, which was enough for the polymer samples. The scanning speed was set to 5°/min.

### 2.4. Design of the Milling Experiments

**Figure 2:**
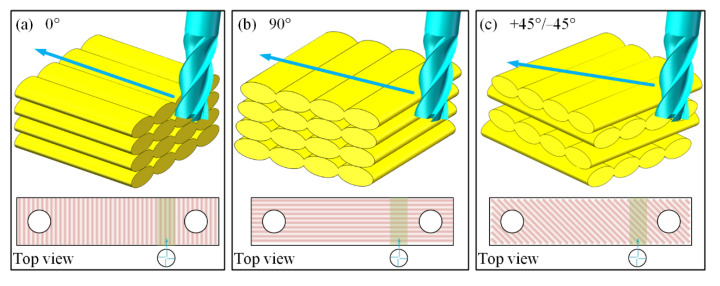
Schematic of milling direction for different RAs: (**a**) 0°; (**b**) 90°; (**c**) +45°/−45°.

Adjusting the RA could generate anisotropic mechanical properties of the 3D-printed parts. Hence, the RA was very likely to influence the milling performance. The three configurations of the end mill feeding direction and the prepared samples have been displayed in Figure 2, i.e., parallel to the filament, perpendicular to the filament, and intersecting with the filament. The top view in Figure 2 illustrates the filament orientation of actual samples and the feeding direction of the end mill. A series of shallow grooves were generated by dry milling with different parameters. The roughness values of the central zone of each groove were compared.

Because of the fact that the wear of tungsten carbide (milling tool material) and the cutting force during machining polymers was very slight compared with the milling of metals, the wear and force of the cutting tool were not investigated in this work. In this research, the depth of the DOC, the SS, and the FRT were selected as the research variables. Among these, the FRT means the feed rate (FR) divided by the tooth quantity of the end mill (i.e., FR/4 in this work). Because the aim of the milling operation was only to remove the superficial rough layer in the semi-finishing or finishing regime, small DOCs (0.1~0.3 mm) were adopted, considering the initial surface roughness and avoidance of the material waste. The selection of the SS and the FRT was based on conventional milling parameters and machine capability. Table 3 summarizes the utilized milling parameters in this work. In the following parts, it can be noticed that the surface morphology was direction-sensitive, which cannot be accurately evaluated by line roughness. Hence, surface roughness (Ra) was adopted to comprehensively evaluate the surface quality after dry milling, which can be calculated by the embedded algorithm in LSCM.

## 3. Results and Discussions

### 3.1. Characterisation of the Initial Surface

**Figure 3:**
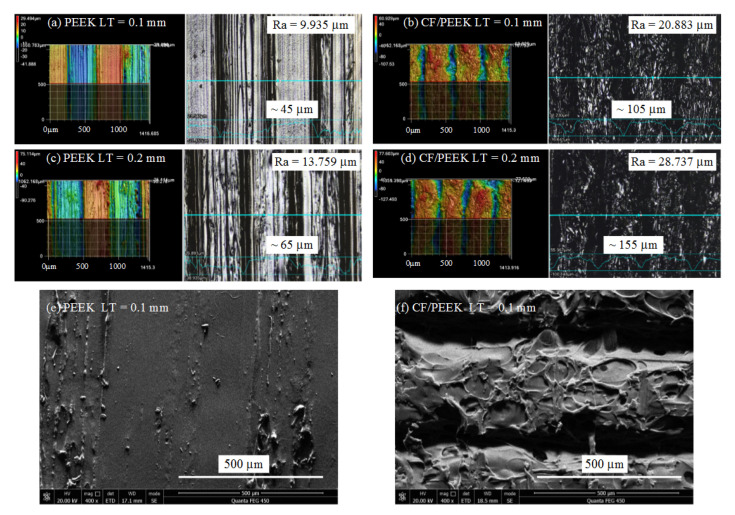
Superficial surfaces of the 3D-printed samples: (**a**) 3D morphology of the PEEK part with LT of 0.1 mm; (**b**) 3D morphology of the CF/PEEK part with LT of 0.1 mm; (**c**) 3D morphology of the PEEK part with LT of 0.2 mm; (**d**) 3D morphology of the CF/PEEK part with LT of 0.2 mm; (**e**) SEM picture of the PEEK part with LT of 0.1 mm; (**f**) SEM picture of the CF/PEEK part with LT of 0.1 mm.

**Figure 4:**
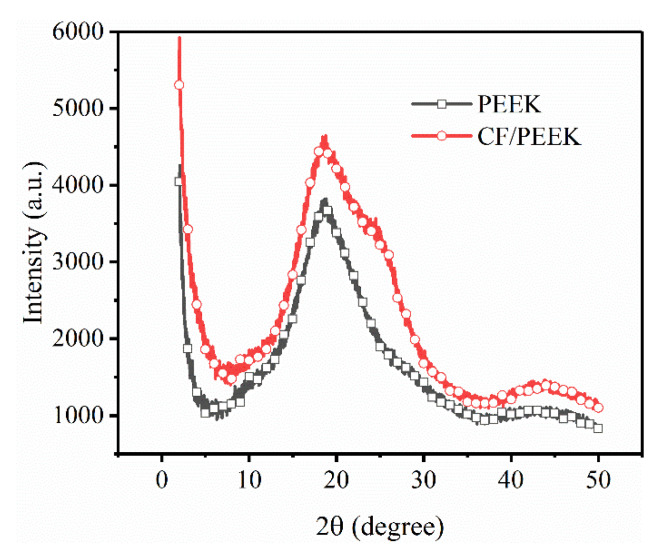
XRD pattern of the PEEK and CF/PEEK samples.

Since this research focused on the removal of the near surface zone, it was important to measure the top surface morphologies. Figure 3 presents the morphologies of the superficial layer of 3D-printed PEEK and CF/PEEK parts under different LTs, together with Ra values. It can be noticed that the fluctuation of the PEEK parts was below 100 μm. The CF/PEEK counterpart revealed rougher morphologies, the fluctuation of which exceeded 100 μm. The SEM pictures (Figure 3e,f) exhibit the details of the deposited filaments. Rugged microstructures can be observed on the deposited CF/PEEK filament. Since crystal structure and morphology can be confirmed by XRD, the amorphous state of the superficial layer was examined by the XRD patterns. In Figure 4, no sharp peaks indicating crystallization can be observed. Only the broad diffuse halo of the amorphous phase can be derived in both the spectra of the PEEK and CF/PEEK samples.

### 3.2. Effects of Printing Parameters

#### 3.2.1. Effects of the RA

**Figure 5:**
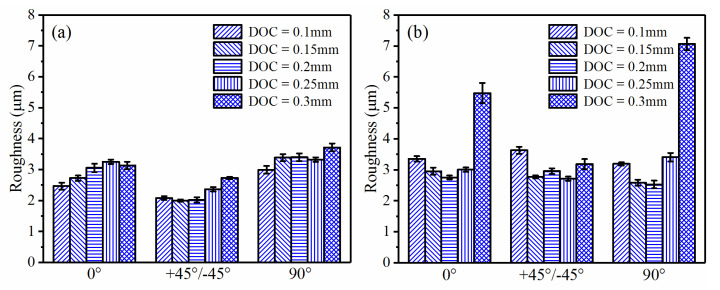
Roughness comparison with different RAs (LT = 0.1 mm, SS = 3000 r/min, FRT = 0.04 mm): (**a**) PEEK; (**b**) CF/PEEK.

Firstly, the effect of the RA was examined under different DOCs. Figure 5 illustrates the changing roughness trend under different RAs. As shown in Figure 5a, when the RA was set to +45°/−45°, the Ra exhibited the lowest value no matter how DOC changed. However, for the case of CF/PEEK in Figure 5b, the Ra exhibited no significant sensitivity to RA, except for the case when the DOC was set to the maximum value, 0.3 mm.

**Figure 6:**
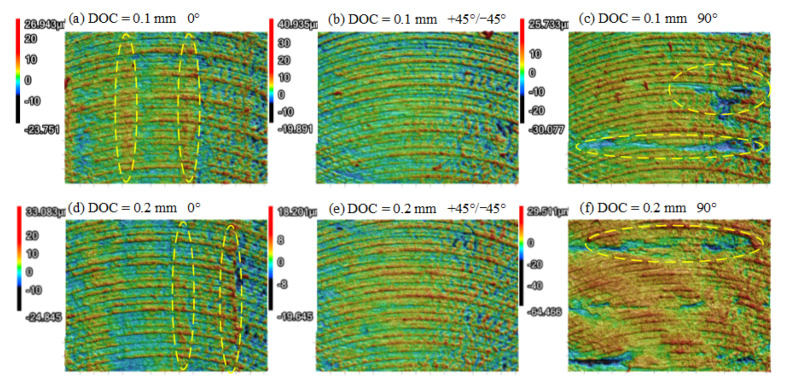
3D morphologies of milled PEEK parts under different RAs: (**a**) DOC = 0.1 mm, RA = 0°; (**b**) DOC = 0.1 mm, RA = +45°/−45°; (**c**) DOC = 0.1 mm, RA = 90°; (**d**) DOC = 0.2 mm, RA = 0°; (**e**) DOC = 0.2 mm, RA = +45°/−45°; (**f**) DOC = 0.2 mm, RA = 90°.

Figure 6 illustrates the morphologies of the PEEK samples machined with the parameters listed in the caption of Figure 5. When the RA was set to 0°, raised ridges were distinct along the same direction of the filament deposition, as typically circled in Figure 6a,d. The ridges resulted in the degradation of Ra. When RA was set to 90°, some parts of the gaps between filaments were filled with the polymers, while other parts of the gaps displayed voids. Because of these voids in the gaps, the roughness indicated a degradation trend. This phenomenon also revealed that the material smearing could increase the surface quality of the 3D-printed polymers. As a comparison, the PEEK samples with an RA of +45°/−45° (Figure 6b,e) exhibited a more uniform and smooth surface. Hence, the feeding direction inclined at 45° to the filament deposition direction was beneficial to better surface quality.

**Figure 7:**
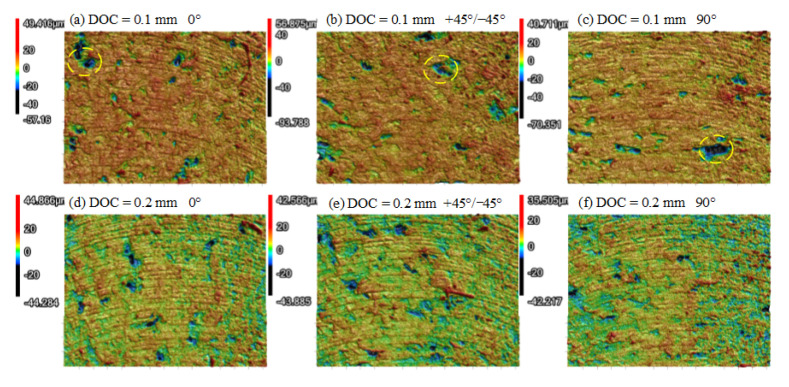
3D morphologies of milled CF/PEEK parts under different RAs: (**a**) DOC = 0.1 mm, RA = 0°; (**b**) DOC = 0.1 mm, RA = +45°/−45°; (**c**) DOC = 0.1 mm, RA = 90°; (**d**) DOC = 0.2 mm, RA = 0°; (**e**) DOC = 0.2 mm, RA = +45°/−45°; (**f**) DOC = 0.2mm, RA = 90°.

The morphologies of CF/PEEK parts under different RAs are shown in Figure 7. Different from the morphologies of the milled PEEK sample in Figure 6, many cavities and defects were discretely presented on the 3D-printed CF/PEEK samples after dry milling, as circled in Figure 7. No traces related to the directional deposited filament could be observed. As a result, the surface roughness was insensitive to RA in terms of Ra. From the perspective of the morphologies, the ductile removal was the main removal method for the PEEK and CF/PEEK samples. However, some brittle removal could be noticed on the CF/PEEK samples, such as the cavities. Similarly, Q. Li et al. [37] reported that the addition of CF decreased the layer-to-layer bonding strength, and significantly influenced the fracture mode of CF/PEEK. They found that semi-brittle fractures also existed in the vertically printed CF/PEEK composites.

#### 3.2.2. Effects of LT

**Figure 8:**
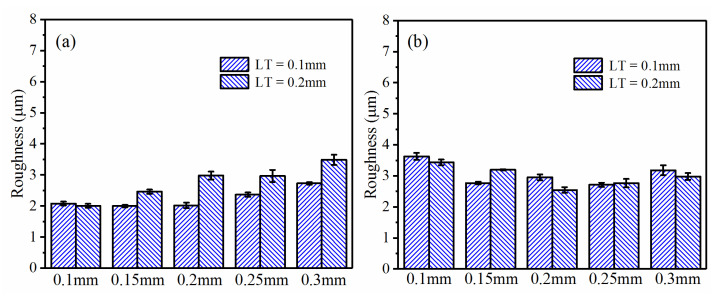
Roughness comparison subject to different LTs (RA = +45°/−45°, SS = 3000 r/min, FRT = 0.04 mm): (**a**) PEEK; (**b**) CF/PEEK.

In FDM, LT plays an important role in printing precision and efficiency for 3D-printed PEEK and its composites. However, the effect of LT on dry milling with a small DOC was still unknown. The samples under RA of +45°/−45° were selected since the surface quality was better at this RA, as indicated above. Figure 8 summarized Ra variation subject to different LTs. For the PEEK samples, the Ra exhibited a degradation trend when LT increased to 0.2mm (Figure 8a). However, for the CF/PEEK samples, there was no definite trend when changing LT, as shown in Figure 8b.

**Figure 9:**
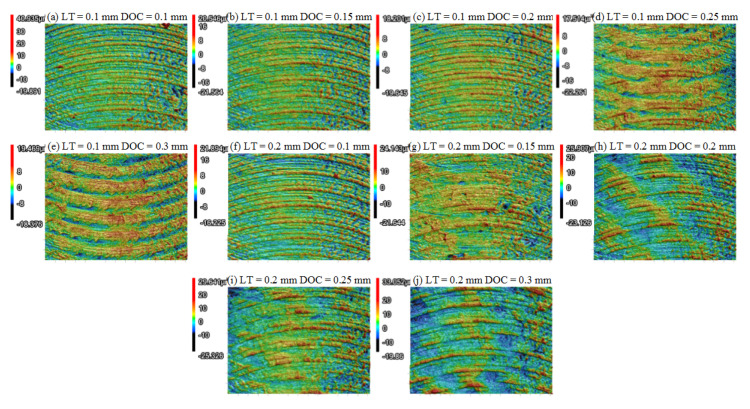
3D morphologies comparison (RA = +45°/−45°, SS = 3000 r/min, FRT = 0.04 mm): (**a**) LT = 0.1 mm, DOC = 0.1 mm; (**b**) LT = 0.1 mm, DOC = 0.15 mm; (**c**) LT = 0.1 mm, DOC = 0.2 mm; (**d**) LT = 0.1 mm, DOC = 0.25 mm; (**e**) LT = 0.1 mm, DOC = 0.3 mm; (**f**) LT = 0.1 mm, DOC = 0.1 mm; (**g**) LT = 0.1 mm, DOC = 0.15 mm; (**h**) LT = 0.1 mm, DOC = 0.2 mm; (**i**) LT = 0.1 mm, DOC = 0.25 mm; (**j**) LT = 0.1 mm, DOC = 0.3 mm.

Figure 9 presents the morphologies under LTs of 0.1 mm and 0.2 mm. When the DOC was 0.1 mm, there was no obvious difference between Figure 9a,f. When the DOC increased to 0.2 mm and above, the morphologies changed significantly. Relatively clear ductile milling traces could be observed when LT was set to 0.1 mm. When the LT was equal to 0.2 mm, raised ridges related to the deposited filament were obvious, resulting in the increase in Ra. From this point of view, when DOC was in a lower regime, the LT only exerted limited influence on the surface quality after dry milling, which can be a criterion for the selection of the DOC.

### 3.3. Effects of Milling Parameters

In this research, the dry milling parameters included the DOC, the SS and the FRT, which are modifiable parameters on commercial machine tools.

#### 3.3.1. Effects of the DOC

The selection of the DOC firstly relies on the surface’s initial fluctuation. In principle, a too-small DOC cannot effectively remove the rough surface layer and enhance the surface quality. A large DOC tends to waste the high-performance polymers. Moreover, cutting force increases with the DOC. Because polymer parts possess lower stiffness, the periodic deformation is more sensitive to the cutting force. Hence, an overlarge DOC may deteriorate the finished surface. Since the DOC is quite crucial for dry milling of the 3D-printed polymers, investigations on the effects of the DOC on 3D-printed PEEK and CF/PEEK have been conducted.

**Figure 10:**
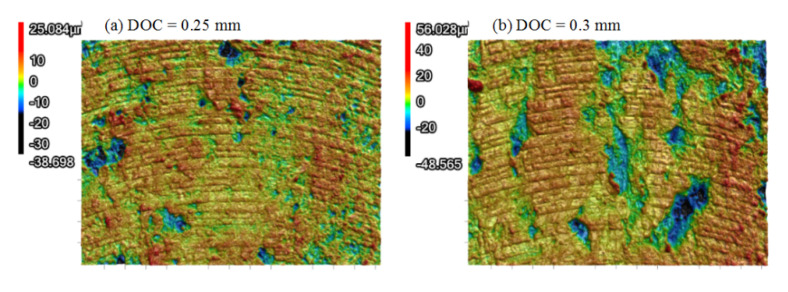
3D morphologies of CF/PEEK samples (RA = 0°, LT = 0.1 mm): (**a**) DOC = 0.25 mm; (**b**) DOC = 0.3 mm.

In the above discussions, DOC has been mentioned as a variable for evaluation of the FDM parameters (RA and LT). For example, the roughness of the PEEK samples in Figure 5a indicated an upward trend with the increase in the DOC in spite of several exceptions. The similar trend can also be found in Figure 8a. For CF/PEEK samples, the roughness exhibited a tendency to decrease first and then increase, as presented in Figure 5b. When the DOC was equal to 0.1 mm, the roughness always presented relatively large values. Since the fluctuation of the superficial deposited filaments exceeded 0.1 mm, the DOC of 0.1 mm may not have been enough for this kind of CF/PEEK sample. When 0.3 mm was applied for the DOC, extremely high roughness values occurred in Figure 5b. The 3D morphologies of CF/PEEK subject to DOCs of 0.25 mm and 0.3 mm were presented in Figure 10. When the DOC was set to 0.3 mm, large strips of polymers appeared on the finished surface, which deteriorated the finished surface quality. Hence, a DOC ranging from 0.15 mm to 0.25 mm was suitable for dry milling of the CF/PEEK superficial zone.

#### 3.3.2. Effects of the SS

**Figure 11:**
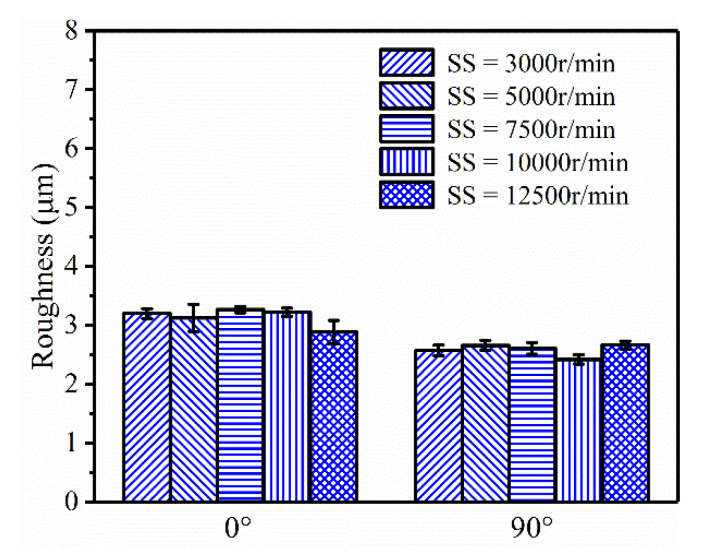
Roughness of PEEK samples under different SS (LT = 0.1 mm, DOC = 0.2 mm, FRT = 0.02 mm).

The SS is a key factor that is often adjusted in practical machining. An overhigh SS tends to significantly increase the temperature of the spindle, which is unfavorable to stable continuous machining. Figure 11 illustrates the roughness variation of the PEEK samples under five progressive SSs at two levels of RA (0° and 90°). However, there was no obvious variation in roughness, although SS changed drastically. The Ra fluctuated between 2.5 μm and 3.5 μm.

**Figure 12:**
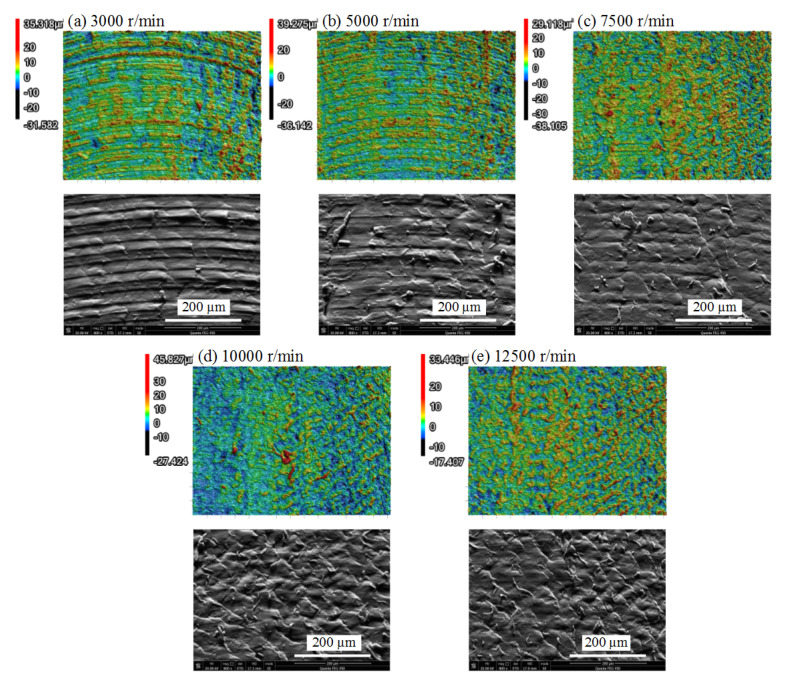
3D morphologies and SEM pictures under different SSs (RA = 0°, LT = 0.1 mm, DOC = 0.2 mm, FRT = 0.02 mm,): (**a**) SS = 3000r/min; (**b**) SS = 5000r/min; (**c**) SS = 7500r/min; (**d**) SS = 10000r/min; (**e**) SS = 12,500 r/min.

However, the morphologies and microstructures under the progressive SS (Figure 12) changed markedly, although there was not much difference in roughness values. When the SS was below 5000 r/min, milling stripes were clear in the 3D morphologies and SEM pictures. With the increase in SS, cataphracted structures appeared gradually until the whole area was filled with the cataphracted structures, as shown in Figure 12e. Ductile removal was still in charge of the cutting process based on the morphologies. XRD results confirmed that no crystallization happened when the SS was set to 12,500 r/min. This morphology change may be ascribed to the tool–workpiece vibration coupling due to the change in SS, which requires further investigation. The surface morphologies can be adjusted by changing the SS, which can be applied to some surface-sensitive occasions.

#### 3.3.3. Effects of the FRT

**Figure 13:**
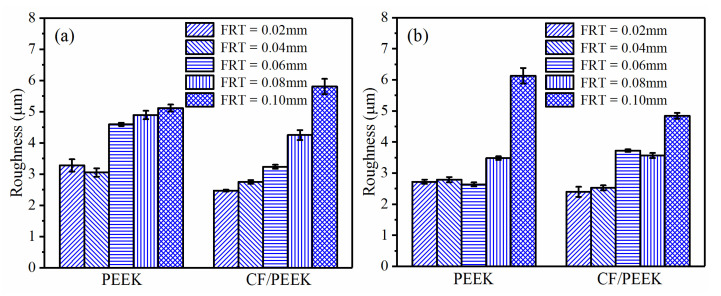
Roughness comparison of PEEK and CF/PEEK samples under different FRTs: (**a**) LT = 0.1 mm, RA = 0°, SS = 3000 r/min; (**b**) LT = 0.1 mm, RA = 90°, SS = 5000 r/min.

The FRT determines the milling efficiency. A higher FRT contributes to accelerating the surface finishing. However, an overlarge FRT also probably aggravates the surface quality of the polymers. Figure 13 compares the roughness under different FRTs. With the increase in FRT, the Ra generally displayed an increasing trend for both PEEK and CF/PEEK samples, which was similar with the metal cutting.

**Figure 14:**
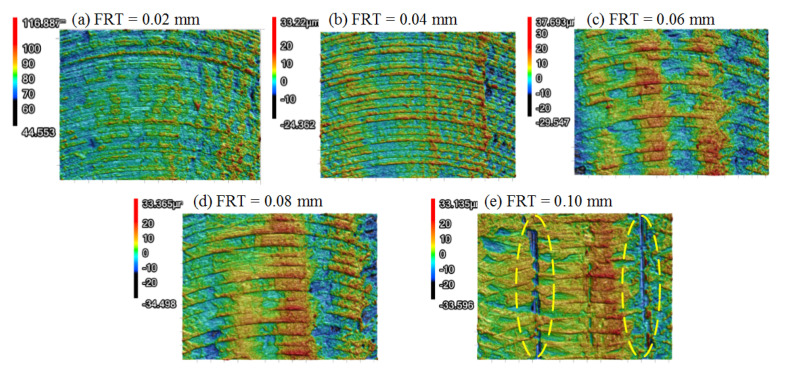
3D morphologies comparison of PEEK samples under different FRTs (RA = 0°, LT = 0.1mm, SS = 3000r/min): (**a**) FRT = 0.02 mm; (**b**) FRT = 0.04 mm; (**c**) FRT = 0.06 mm; (**d**) FRT = 0.08 mm; (**e**) FRT = 0.10 mm.

Figure 14 illustrates the 3D morphologies of PEEK samples under different FRTs. When the FRT was set to 0.02 mm and 0.04 mm, the ridges along the filament were obscure. When the FRT increased to 0.06 mm and 0.08 mm, the ridges became obvious because of incomplete removal. When the FRT was set to 0.1 mm, the crevice between the filaments was quite clear, as circled in Figure 14e. Based on the morphology evolution of PEEK samples, it can be deduced that when the FRT was in a small regime, the smearing effect was beneficial to fill the crevice between the deposited filaments. When a large value was set to the FRT, the smearing effect was weakened, and the crevices were exposed.

**Figure 15:**
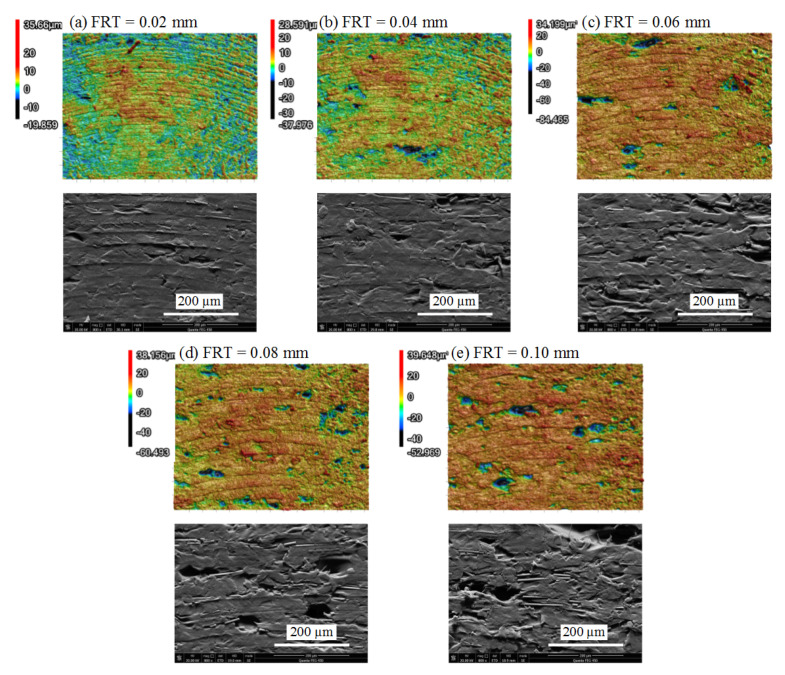
3D morphologies comparison of CF/PEEK parts under different FRTs (RA = 90°, LT = 0.1 mm, SS = 5000 r/min): (**a**) FRT = 0.02 mm; (**b**) FRT = 0.04 mm; (**c**) FRT = 0.06 mm; (**d**) FRT = 0.08 mm; (**e**) FRT = 0.10 mm.

3D morphologies and SEM pictures of CF/PEEK samples subject to different FRTs are presented in Figure 15. It could be noticed that the cavities increased gradually with the growth of FRT. When the FRT was set to 0.02 mm and 0.04 mm, the morphologies exhibited compact plastic deformation. When the FRT was set to 0.06 mm and above, surface degradation occurred after dry milling. There were fewer plastic forming marks compared with the PEEK samples presented above. In Figure 15d,e, the bare carbon fibers were embedded on the finished surface without coverage of the PEEK matrix, indicating a serious surface degradation.

Based on the above analysis, the FRT significantly influenced the finished surface quality of PEEK and CF/PEEK parts. For PEEK, the excessive FRT reduced the smearing effect, deteriorating the surface quality. For CF/PEEK, the voids were more likely to form when the FRT was set to a high value. Considering the roughness values and surface morphologies, an FRT of 0.02~0.04 mm was reasonable for the current conditions, when the DOC was 0.2 mm.

### 3.4. Comparison between 3D-Printed PEEK and CF/PEEK during Dry Milling

**Figure 16:**
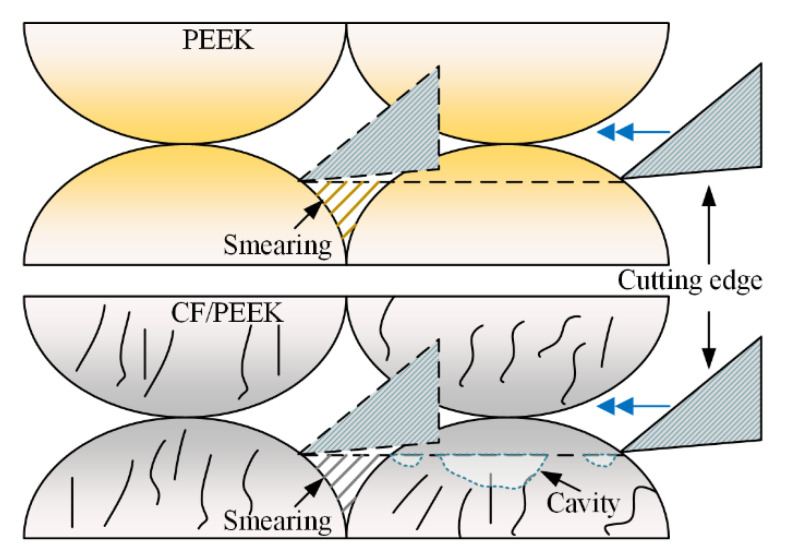
Schematic of the cutting of 3D-printed PEEK and CF/PEEK samples.

The schematic of the cutting of 3D-printed PEEK and CF/PEEK samples has been illustrated in Figure 16. Under the compressive stress, the polymer exhibited toughness, and plastic slipping occurred. These slipping polymers filled the crevice between the filaments along the cutting direction, as displayed in Figure 16. This kind of smearing effect enhanced the surface roughness after dry milling. The introduction of CF led to an increase in brittleness and a decrease in elongation at break, as mentioned in Table 1. Hence, semi-brittle removal, such as cavities, occurred when the DOC or the FRT were set within an overlarge regime.

**Figure 17:**
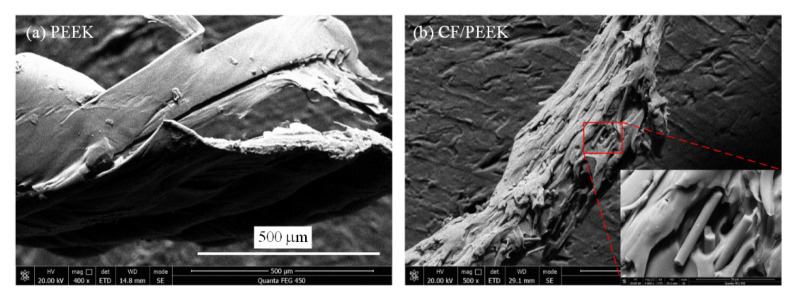
Chip morphologies of PEEK and CF/PEEK samples.

When milling the amorphous polymers, the cutting edge pushed the material to one side under compression and distortion, finally forming the chips. However, the chips always adhered to the edges of the shallow groove, which was also noticed in the work of Y. Yan et al. [38]. Figure 17 presents the chip morphologies of PEEK and CF/PEEK after dry milling. The smooth but distorted chips of the PEEK sample can be observed in Figure 17a. The chips of CF/PEEK present a rough surface with some bare carbon fibers (Figure 17b). When the tool path during the milling process was partially overlapped, these adhered chips or burrs could be removed completely.

In order to further reveal the smearing effect, an FEM model of the cutting of the deposited PEEK filament was presented based on the Johnson–Cook (JC) model [39]. The JC model was widely used in ductile metal alloys, which can also be utilized for analyzing the dynamic behavior of polymer materials:

(1) $$\bar{\sigma }\left({\bar{\varepsilon }}^{p},{\dot{\bar{\varepsilon }}}^{p},T\right)=\left[A+B\cdot {\left({\bar{\varepsilon }}^{p}\right)}^{n}\right]\left[1+C\cdot \ln\left(\frac{{\dot{\bar{\varepsilon }}}^{p}}{{\dot{\bar{\varepsilon }}}_{0}^{p}}\right)\right]\left[1-\Theta^{m}\right]$$

(2) $$\Theta =\frac{T-T_{0}}{T-T_{m}}$$

where ${\bar{\varepsilon }}^{p}$ is the strain hardening, ${\dot{\bar{\varepsilon }}}^{p}$ is the strain rate sensitivity, *A* and *B* are material constants, *n* is the strain hardening exponent, *C* is the strain rate sensitivity parameter, *m* is the temperature sensitivity, *T*_0_ is the initial temperature, and *T_m_* is the melting temperature.

The temperature increase during the cutting process was also considered:

(3) $$\Delta T\left({\bar{\varepsilon }}^{p},{\dot{\bar{\varepsilon }}}^{p},T_{0}\right)=\frac{\beta }{\rho \cdot C_{p}}\int_{\varepsilon }^{{\bar{\varepsilon }}^{p}}\bar{\sigma }\left({\bar{\varepsilon }}^{p},{\dot{\bar{\varepsilon }}}^{p},T\right)d{\bar{\varepsilon }}^{p}$$

where *T* is the current temperature, *T*_0_ is the room temperature, *ρ* is the density of the material, *β* is the Quinney–Taylor heat fraction coefficient, and *C_p_* is the specific heat.

The fracture model included ${\bar{\varepsilon }}^{p}$, ${\dot{\bar{\varepsilon }}}^{p}$, and T dependencies based on the JC fracture model. Failure was assumed when *D* exceeded some value. The *D* parameter was the summation of all increments if deformation occurred. The evolution of *D* follows:

(4) $$D\left({\bar{\varepsilon }}^{p},{\dot{\bar{\varepsilon }}}^{p},T\right)=\sum \frac{\Delta {\bar{\varepsilon }}^{p}}{{\bar{\varepsilon }}_{f}^{p}\left({\dot{\bar{\varepsilon }}}^{p},T,\sigma^{*}\right)}$$

(5) $${\bar{\varepsilon }}_{f}^{p}=\left[D_{1}+D_{2}\cdot \exp\left(D_{3}\cdot \sigma^{*}\right)\right]\left[1+D_{4}\cdot \ln\left(\frac{{\dot{\bar{\varepsilon }}}^{p}}{{\dot{\bar{\varepsilon }}}_{0}^{p}}\right)\right]\left[1+D_{5}\cdot \Theta \right]$$

where $\Delta {\bar{\varepsilon }}^{p}$ is an increment of accumulated equivalent plastic strain that occurs during an integration cycle, and ${\bar{\varepsilon }}_{f}^{p}$ is the critical failure strain level. *D_i_* are failure constants. The constitutive parameters and fracture model parameters can refer to ref. [39]. 

The JC model is generally pre-implemented in FEM software, such as ABAQUS/explicit, on which a 2D finite element model for the simulation of cutting of 3D-printed PEEK was developed with the above parameters. The geometry of the workpiece was 500 μm × 150 μm, which simulated the cross section of a deposited PEEK filament in a certain layer. Hence, only the bottom was fixed, and the other three sides were set to free boundaries. The radius of the cutting edge was set to 20 μm, which was a typical value for the conventional end mill. The cutting speed was set to 1 m/s, and the depth of cut was set to 30 μm.

**Figure 18:**
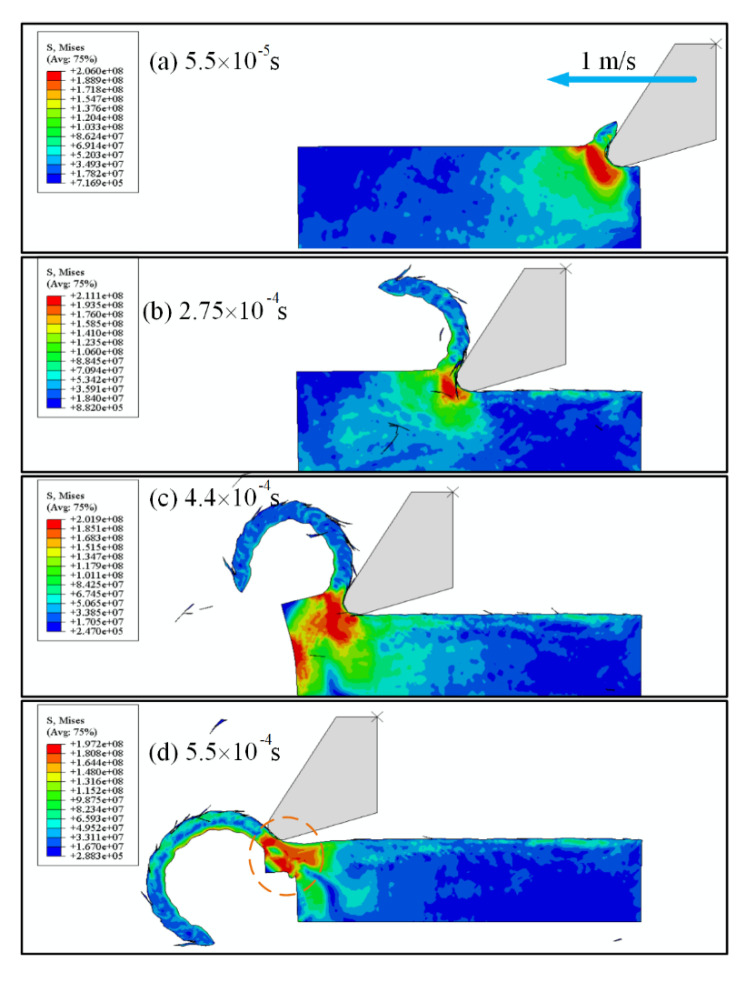
Chip formation during cutting the deposited filament.

The chip formation process with time is illustrated in Figure 18. When the cutting edge continuously contacted with the workpiece, the continuous chip formed under the condition of shear slip. When the cutting edge left the filament, because of the lack in support strength at the very edge of the filament, the polymer material continuously slipped and rotated about some point (Figure 18d). It can be noticed that there was no separation of the chip and the workpiece under current conditions. The outstretched polymer material, as circled in Figure 18d, could fill the crevice between the deposited filaments (the smearing effect), which was observed in the above experiments.

### 3.5. Comparison of Additive–Subtractive Manufacturing of PEEK and CF/PEEK

**Figure 19:**
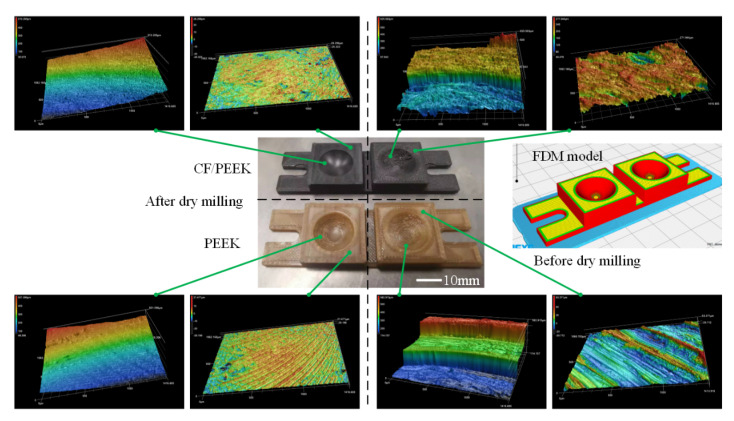
Comparison of additive–subtractive manufacturing of PEEK and CF/PEEK parts: before dry milling (**right**) and after dry milling (**left**).

The effects of printing parameters and milling parameters were evaluated for the dry milling of PEEK and CF/PEEK. In this part, a comparison of additive–subtractive manufacturing of PEEK and its composites is presented. Firstly, two of the same structures consisting of plane and curved surfaces were fabricated by FDM of PEEK and CF/PEEK, as shown in Figure 19. The milling parameters are listed in Table 4. The selection of parameters for the ball mill also referred to the above analysis. Figure 19 compared the morphologies before and after dry milling. The coarse accumulation of the molten materials could be observed especially on the concave spherical surface. After one-time dry milling of the surface layer with the given parameters, shinning surfaces could be achieved without any adhered chips. For the PEEK sample, because of its translucent property, the layering state could be noticed, in addition to the reflective surface. From the perspective of micro morphologies captured by LSCM, the staircase effects were removed completely. This case study indicated that dry milling after FDM could be a promising post-processing method for high-performance polymers, which can be integrated into high-performance polymer 3D printers.

## 4. Conclusions

In order to enhance the surface quality of 3D-printed PEEK and CF/PEEK parts to meet the critical requirements of high-temperature molds, medical implants, and precision components, this work investigated dry milling the fluctuant exterior surface of the 3D-printed amorphous PEEK and CF/PEEK parts before heat treatment. The co-dependency between 3D-printing parameters (the raster angle and the layer thickness) and the milling parameters (the depth of cut, the spindle speed, and the feed rate per tooth) for the PEEK and CF/PEEK samples was studied through experiments. To be specific:

- Both the raster angle and the layer thickness could affect the dry milling performance. The raster angle of +45°/−45° and a thinner layer facilitated better surface qualities.
- An overlarge depth of cut and feed rate per tooth were likely to deteriorate the finished polymer surface. The spindle speed could influence the morphologies without significant changes in roughness values. In the future, side milling will be investigated to expand the scope of dry milling.
- The smearing effect can enhance surface leveling and decrease defects for both PEEK and CF/PEEK samples. The shear slip mechanism of the smearing effect was revealed by a simulation model.
- A demonstration was put forward to verify that dry milling of 3D-printed PEEK and CF/PEEK parts with suitable parameters can significantly improve the surface quality and decrease defects, such as filament accumulation. This dry milling method can be extended to the other 3D-printed amorphous polymers and can even be integrated to 3D printers for high-performance polymers.

## Figures and Tables

**Table 1:** Material properties of the experimental PEEK and CF/PEEK.

Item	Test Standard	PEEK	CF/PEEK
Tensile strength (MPa)	ISO 527	99.9	87.4
Young’s modulus (MPa)	ISO 527	3738	5193
Elongation at break (%)	ISO 527	9.1	2.9
Bending strength (MPa)	ISO 178	147	168.6
Flexural modulus (MPa)	ISO 178	3612	6338
Notch impact strength (KJ/m^2^)	ISO 179	7.1	9.7
Heat distortion temperature (°C)	ISO 75, 1.8MPa	152	315
Glass transition temperature (°C)		143	143
Melting point (°C)		343	343

**Table 2:** Parameters for FDM processing.

Items	Parameters
Filaments	PEEK, CF/PEEK
Printing speed (mm/s)	50
Nozzle diameter (mm)	0.4
Nozzle temperature (°C)	400
Build plate temperature (°C)	130
Build plate treatment	Use the frosted glass plate. Apply PVP glue.
Cooling fan speed	50%
Layer thickness (mm)	0.1, 0.2
Raster angle	0°, 90°, +45°/−45°

**Table 3:** Parameters for the milling experiments.

Items	Parameters
DOC (mm)	0.1, 0.15, 0.2, 0.25, 0.3
SS (r/min)	3000, 5000, 7500, 10,000, 12,500
FRT (mm)	0.02, 0.04, 0.06, 0.08, 0.1

**Table 4:** Parameters for dry milling of the plane and curve surfaces.

Tool	Feature	DOC (mm)	SS (r/min)	FR (mm/min)	Overlapping
End mill (∅4)	Plane	0.2	5000	400	50%
Ball mill (∅2)	Curve	0.25	8000	400	50%

## Data Availability

The data presented in this study is openly available.
